# Dr. M'Whirter's Case of Delirium

**Published:** 1807-08

**Authors:** T. M'Whirter

**Affiliations:** Physician to the Newcastle Dispensary. Newcastle upon Tyne


### *To the Editors of the Medical and Physical Journal.*

GENTLEMEN,

YOUR having honoured with insertion, in your Number for February, the hasty remarks on burns and scalds, bearing my initials, is an inducement to address you again. It has given me pleasure to see the subject so ably investigated, and to find that the cooling treatment, in the early stages, has been so often and successfully employed; while I hope that the opposite plan will not, upon suitable occasions, be neglected. There is a consequence of extensive burns, which I presume is of rare occurrence, and has not attracted much notice. As I had lately an opportunity of seeing it, I shall briefly make it known.

### CASE SIMILAR TO BRAIN FEVER, SUCCEEDING A BURN.

A middle aged man, who, with many others, had been universally scorched by the firing of a coal-mine in this neighbourhood, and who had been treated according to Dr Kentish's method, for eight or ten days, with apparent success, insomuch that a great part of the skin had been reproduced, became suddenly delirious. He seemed very restless; his pulse beat 130 weak strokes in a minute; his tongue was perfectly clean, his feet rather cold, his skin cool. The resemblance of his case to the disease, here called the Brain Fever, which attacks men that drink by the week or fortnight and give it over suddenly, suggested a similar treatment. He was ordered a pint of Port wine warmed, and with the addition of spices, to be taken as expeditiously as possible, and so soon as medicine could be sent, to take a draught with thirty drops of laudanum and a drachm of æther, every two or three hours; hours; which were to be gradually reduced in strength after the delirium had subsided, and sleep had been procured.

He was directed to have strong soups as food, till he could return to his common diet..

He began this plan in the evening, and towards morning he fell asleep, and awoke perfectly well and unconscious of what had been passing. His remedies were gradually withdrawn. I have seen but one case of Brain Fever (its nosological name I cannot easily find) the symptoms of which were very similar, and yet not so severe, while their cause was much more easily ascertained. I shall mention them, as the disease, it is to be hoped, is not frequent, and as the mistaking of it might lead very readily to fatal practice.

### Case of Brain Fever.

A middle aged man, who had been intoxicated for a week, indeed had tasted nothing but spirits, was seized in the night with convulsions. When I saw him, he was vomiting bile, and nothing of the simple kind offered, had remained in the stomach. He was directed to have a strong tumbler of brandy and water, and some warm beef tea; the former twice or thrice in the day, the latter as often as he could take it.

Having been very costive a brisk purgative was necessary, and the above plan was intended to prevent the attack of fever, which was to be otherwise expected. He had an opiate at night. He was perfectly well next morning, and going about as usual. His friends were cautioned, not at once to withdraw his spirits and water.

They gave him some that day and he was well; but next day he got none, and by the evening was seeing white ghosts, and a variety of invisible beings. When spoken to, his answers were for a moment rational, but wandered in the next to a thousand different subjects, mostly marked by gaiety.

He talked incessantly. Pulse 30, intermitting at times, not remarkble in point of strength. Tongue perfectly clean; skin cool; countenance expressive of a thousand feelings in a minute; eyes remarkably glistening and animated. He was ordered a draught of thirty drops of laudanum, which had not been used the night before, and the shops had been shut, so that it could not be made stronger. He had a glass of spirits and water and was put to bed. He at first had been more quiet, but did not not sleep; but about one in the morning he got up, insisted upon going out, and became ungovernable.

He sometimes saw watchmen coming to drag him away, at others made merry with his friends, visible only to himself.

He refused to take any thing, and to return to bed, and struggled and shouted violently, when compulsion was used. He attempted to cut his throat, and to get out at the window. Just when most outrageous, I dashed a pitcher of water upon his face. He shrunk into a corner, begging piteously for mercy, and promising to do any thing. Having procured laudanum, he took one hundred drops, and was ordered fifty every two hours, till he should sleep. Beef tea, &c. were ordered as before. He slept at five in the morning for four hours. The laudanum was reduced to thirty drops every two hours during the intervals of sleep. He slept some hours in the afternoon, and all night, and was quite well next day.

The laudanum was continued in gradually reduced doses for three days, with 30 drops every night at bed-time.

The bowels were opened regularly. He was much better than for some months previously, had regained a good appetite, and for a week or two had abstained from every liquor, except water.

### Remarks.

It will surely occur, that there is a striking similarity between these two cases, though I did not see the former so early after the attack. The pain of burning and turpentine having been applied to nearly the whole surface, must have been a high stimulus; its sudden subsidence, like the sudden abstaining from spirits, seems fairly attributable as the cause of the subsequent fever, in the former case as well as in the latter. Experience, independently of theory, has approved of the practice detailed. There are individuals, I am credibly informed, in this neighbourhood, who have survived seven attacks of Brain Fever.

I have been also well assured, that where blood-letting, blistering, and abstinence have been prescribed, the patients have to a man, died. I saw a gentleman about a year ago, who would, probably, have illustrated the truth of the above information.

### Case of Paralysis, &c. from suddenly leaving off Drinking.

He was middle-aged, immensely corpulent, had drank two bottles a day of brandy, for a fortnight; upon leaving it it off, at once, had become paralytic of his hands and feet. His medical attendants had let blood (I believe) but at any rate had purged him freely, and recommended abstinence. He had become suddenly and alarmingly worse. He lived fifteen miles off, and I did not see him till midnight. His breathing was greatly oppressed; his extremities cold; his pulse fluttering; his face blue; and he was shivering. I ordered him a pint of warm brandy immediately; by morning he had taken a bottle and a half.

When medicines could be procured, he was treated as in the former instances, and recovered, and remains temperate. It would seem that the human system will bear no sudden reduction of high stimulant powers with impunity; and, therefore, that we ought to be very inquisitive as to the causes of such peculiar and rare disorders, and particularly, as it may sometimes be attempted to conceal them. I shall be happy if these sketches prove sufficient resemblances to make known the originals, should they be introduced to any of your readers, as strangers.

I fear you will think I have been very diffuse; but I leave you the liberty of dealing with me as you please.

I am, &c.

T. M'WHIRTER, M.D. Edin.
Physician to the Newcastle Dispensary.

